# Sustained attention assessment of narcoleptic patients: two case reports

**DOI:** 10.1590/S1980-57642009DN20400020

**Published:** 2008

**Authors:** Mirleny Moraes, Barbara A. Wilson, Sueli Rossini, Kátia Osternack-Pinto, Rubens Reimão

**Affiliations:** 1 Neuropsychologist, Sleep Medicine Advanced Research Group, Division of Clinical Neurology, Hospital das Clínicas, University of São Paulo Medical School, São Paulo SP, Brazil.; 2 Senior Scientist, Clinical Psychologist PhD, MRC Cognition and Brain Sciences Unit, Cambridge and Director of Research at Oliver Zagwill Centre, Ely, UK.; 3 Psychologist, PhD, Sleep Medicine Advanced Research Group, Division of Clinical Neurology, Hospital das Clínicas, University of São Paulo Medical School, São Paulo SP, Brazil.; 4 Neuropsychologist, PhD, Division of Psychology, Hospital das Clínicas, University of São Paulo Medical School, São Paulo SP, Brazil.; 5 Neurologist, MD, PhD, Sleep Medicine Advanced Research Group, Division of Clinical Neurology, Hospital das Clínicas, University of São Paulo Medical School, São Paulo SP, Brazil.

**Keywords:** sleep, sleep disorders, excessive daytime sleepiness, narcolepsy, sustained attention, d2 Test, neuropsychology

## Abstract

Narcolepsy is a sleep disorder characterized by uncontrollable REM sleep attacks which alter the patients wake state and can lead to difficulties in attention aspects, such as maintaining attention when performing activities or tasks. This study aimed to evaluate sustained attention performance of two narcoleptic patients on the d2 Test, Epworth Sleepiness Scale (ESS), Pittsburgh Sleep Quality Index (PSQI) and Hamilton Rating Scale for Depression (HAM-D). Results showed that the maintenance of attention was associated with a slowing of the target symbols processing function in visual scanning with accuracy in task performance. A high degree of excessive sleepiness was observed, along with mild and moderate degrees of depressive signs and symptoms. One subject also presented with a nocturnal sleep disorder which could represent an important factor affecting attentional and affective capacity.

## Abstract (pt)

A narcolepsia é um distúrbio do sono caracterizado por ataques diurnos incontroláveis de sono REM, que alteram o estado de vigília dos pacientes, podendo levar a dificuldades na expressão de aspectos da atenção, como a sua manutenção durante a execução de atividades ou tarefas. Este estudo buscou avaliar a atenção sustentada de dois pacientes com narcolepsia através do Teste d2, da Escala de Sonolência Epworth, do Índice de Qualidade de Sono de Pittsburgh (IQSP) e da Escala de Hamilton para Avaliação da Depressão (HAM-D). Os resultados revelaram que a manutenção da atenção mostrou-se associada ao alentecimento no processamento dos símbolos-alvo na sondagem visual, com precisão na execução da tarefa. Houve presença de sonolência excessiva em nível elevado e sinais e sintomas depressivos em grau leve e moderado. Um dos sujeitos apresentou também prejuízo do sono noturno, podendo ser este mais um fator importante na expressão da capacidade atencional e afetiva.

Narcolepsy is an intrinsic sleep disorder that causes excessive daytime sleepiness (EDS),^1^ characterized by sudden daytime REM sleep attacks which can occur at any time but mostly in monotonous situations. Cataplexy, sleep paralysis and hipnagogic hallucinations may also occur. The prevalence of narcolepsy is 1:40,000 with onset commonly in the second or third decade of life, and chronic progression.

The first description of the disturbance was in 1880 by Jean Baptiste Edouard Gelineau, but only recently (1998)^2^ has it been possible to identify the presence of deficit in the production of hipocretin I and II neurotransmitter (orexin) in the lateral hypothalamus as the underlying cause of narcolepsy.

The symptomatology of this disease severely affects the quality of life of patients in social,^3^ psychological^4^ and neuropsychological^5-11^ domains of daily living. Studies investigating the impact of narcolepsy on neuropsychological functions remain scarce. Patients complain of difficulties in memory for recent events and in maintaining attention for long periods, especially in monotonous situations.^7-9^

Attention studies in groups of narcoleptic patients published to date, have revealed tonic alertness (vigilance), flexible and divided attention impairments and increased latency responses compared to controls.^10^

Sustained or concentrated attention is the individual capacity to maintain focus of attention on a given stimulus for a period of time.^12,13^

The d2 Test is one of the neuropsychological tests used both internationally and within Brazil to evaluate sustained attention, owing to its high sensitivity and accuracy in this kind of attention assessment. It was devised to measure the ability to drive, an activity which requires rapid and accurate observation of details by the driver,^1^ and seeks to assess sustained attention and visual scanning skills,^15^ having been validated in Brazil since 1990.

We now report the assessment of two outpatients diagnosed with narcolepsy, followed at the Neurology Department outpatient clinic of São Paulo University Medical School (HC-FMUSP). This evaluation is part of a larger project by the authors on narcolepsy to be published elsewhere.

This study entailed individual testing as follows:

- The d2 Test to assess sustained attention;^14^
- The Epworth Sleepiness Scale (ESS) that assesses presence of excessive daytime sleepiness;^16^
- The Pittsburgh Sleep Quality Index (PSQI) that evaluates sleep efficiency, classifying subjects as good (≤5 points) or poor sleepers (>5 points);^17^
- The Hamilton Rating Scale for Depression (HAM-D) which indicates the presence of depressive signs and symptoms.^18^

Assessing the attention and other cognitive functions of patients with narcolepsy involves the devising of proposals for neurological rehabilitation to “reduce the impact of their problems in everyday life, and achieve their optimum level of well being”.^19^

## Case reports

This evaluation is part of a larger project on narcolepsy approved on June 2007 by the Ethics Committee of the Institution, Hospital das Clínicas of University of São Paulo Medical School under Research Protocol #0455/07. All patients signed the Informed Consent Term at the beginning of the evaluation.

### Case 1

Involved a young man aged 27y7m, bachelor (living with a girlfriend), with incomplete Engineering degree, who was an English teacher with a high economic level (Table 1).

**Table 1:** Raw and scaled scores of subject 1 on the ESS, PSQI, HAM-D scales and d2Test.

ESS	PSQI	HAM-D		d2 Test				
				TS	TE	TS-E	E%	FR
17	7	18	Scores	386	16	370	4.1	14
			Percentile	25	-	30	50	50

TS, total raw score; TE, total errors; TS-E, total score minus errors; E%, percentage errors; FR, fluctuation rate.

At 17 years he presented with EDS, with gradual worsening of symptoms. At 20 years, he began to manifest insomnia symptoms, and sought medical assistance which yielded an inconclusive diagnosis. He also started to manifest cataplexy, sleep paralysis episodes and memory difficulties. The patient discontinued his degree course because of excessive sleepiness.

He was diagnosed 7 years after first symptoms, and is currently on a course of metilfenidate (4 tabs. 10 mg a day) and clomipramine (4 tabs. 25 mg a day) medication. He has complained of attention difficulties (loss of focus) and immediate memory problems.

### Case 2

Involved a young woman aged 23y4m, bachelor, holding a degree in Letters, who worked as a secretary (Table 2).

**Table 2:** Raw and scaled scores of subject 2 on the ESS, PSQI, HAM-D scales and d2Test.

ESS	PSQI	HAM-D		d2 Test				
				TS	TE	TS-E	E%	FR
22	5	13	Scores	380	13	367	3.4	10
			Percentile	20	-	20	30-40	75-80

TS, total raw score; TE, total errors; TS-E, total score minus errors; E%, percentage errors; FR, fluctuation rate.

The symptoms manifested at 16 years, when attending high school and had begun working. She reported uncontrollable sleep episodes during the day, attributing the symptoms to fatigue, until she met a person who had similar symptoms and began researching the illness.

After neurological consultation, she underwent polysomnography which showed negative results for sleep disorders. Because of lack of a conclusive diagnosis she received treatment for anxiety. The psychiatric evaluation indicated “escape from reality”, and antidepressant medication was prescribed.

Akin to Case 1, seven years after symptoms onset, she was diagnosed with narcolepsy. She was placed on Benzedrine sulfate (2 tabs 5 mg a day).

She had complained of difficulties in focusing attention, drifting from one activity to another and forgetting the previous activity. She did not retain movie story lines or jokes but reported general improvement in various functions after medication, including the reading of texts, hitherto hampered because of excessive sleepiness.

Tables 1 and 2 show the results of our two patients separately , for the instruments used to assess sustained attention.

On the d2Test subject 1 achieved 25% of expected performance for age and level of education on total target symbols processed (TS), according to the Brazilian standards. This figure indicates that speed was low for activities that required sustained attention, predominantly due to omission errors: 12 against 2 errors, evenly distributed throughout the lines and with a fluctuation rate within expected average.

It is possible that the subject made excessive effort to sustain concentration during visual scanning of symbol targets, resulting in greater slowness in implementing the task where an indication consistent with this proposition was the fact that, after finishing the test, the patient complained of intense dizziness.

Impaired sleep efficiency according to PSQI indexes as well as the presence of narcolepsy-related EDS, could have been factors influencing test results and attention quality.

The amount of target symbols processed by subject 2 (TS) and global performance (TS–E: total number of items processed minus errors) on the d2 Test was considerably lower than normal for age and level of education.

Predominant omission errors: 12 against 1 errors occurred from the second half the test, precisely where there was a peak of oscillation, that is, on the line where the patient considered the lowest number of items, although overall fluctuation rate remained stable throughout lines.

Speed associated with the ability to maintain attention in the implementation of the task was impaired.

EDS level was high, but nocturnal sleep quality was good, where this may represent a mitigating factor for the symptoms of narcolepsy.

## Discussion

Both cases fell within the 20–30y age range and had good educational and economic levels. They presented with a high degree of EDS, signs and symptoms of depression at mild and moderate levels, as shown in their results on the HAM-D, and obtained TS–E scores on the d2 Test which were considerably lower than expected for age group and level of education compared to the normative sample for the Brazilian population.

The first case was classified as a “poor sleeper” according to the PSQI indexes that could prove relevant factor for the presence of mild and moderate signs and symptoms of depression and overall d2 Test performance. The patient, also showed physiological symptoms (dizziness) and tiredness following the d2 test. The chronic use of clomipramine, used for cataplexy, could also be considered while a larger number of patients needs to be evaluated for further conclusions to be drawn.

Two subjects committed significant omission errors but much fewer addition errors, suggesting attention deficits or possible fluctuation of alertness state during visual search for target letters in the line, generating lapses of attention, rather than a difficulty in visual discrimination that could have led to the marking of distractor symbols, although the analysis of the Fluctuation Rate (FR) across lines revealed rates greater than or equal to average.

Taken together, data on reduced number of items processed by two subjects coupled with the low number of errors showed that accuracy in marking the correct symbols was related to a decrease in speed of task execution. This is in line with data from Naumann, Bellebaum and Daum^20^ who used various tests, including the d2Test, to assess sustained attention in 15 patients with narcolepsy and compare them to controls, and found that patients processed fewer items than controls within the same time limit, indicating a slowing of processing but with equal quality of performance, that is, a tendency to make less errors than controls, resulting in accuracy-speed correlation.

Several previous reports on cognitive function in narcoleptic patients have shown that more complex tasks, which measure executive functions, are the most susceptible to drowsiness^21,22^ that is, decline in performance may be initially offset by an increase in attention resources, but above a critical level of sleepiness, it can only be reversed by recuperative sleep.^11,22^ Also, for tasks such as the short d2 Test (approximately 5 minutes), patients are able to offset the levels of fluctuation of vigilance by using strategies to avoid sleep attacks^20^ that may overburden the attention network and thus generate slowed scanning and tiredness during and after the task, as demonstrated by case 1.

It is well known that hipocretin neurons have large projections throughout the Central Nervous System^2^ and operate in some coincident regions in the Ascending Reticular Activating System (locus coerulus, for example), responsible for executive control of attention (holding the modulation of attention, regardless of modality of the attended sensory stimulation). The reduction of this neuropeptide in narcoleptic patients influences alertness level, compromising some attention network sub-components and placing greater burden on them in activities which require sustained levels of attention, impacting daily activities of these patients.

Future research in this field could entail comparative studies involving longer tests such as the CPT-II. Further studies could also compare other disorders that also present decreased attention levels such as Attention Deficit-Hyperactivity Disorder.

In conclusion, the deficit in sustaining attention was linked to slowed processing of target symbols by visual scanning, whilst accuracy was attained in the task performance. A high degree of excessive daytime sleepiness was present. This impairment points to the need for future research involving a greater number of narcoleptic patients to possibly confirm this result. The value of this study is alerting clinicians (including clinical neurologists) to the need to evaluate and treat daytime sleepiness and address the impact of impairment on the daily lives of these patients including work, and academic performance.
